# Pan-American Medical Congress

**Published:** 1892-12

**Authors:** 


PAN-AMERICAN MEDICAL CONGRESS.—The JOURNAL acknowledges the receipt of the official preliminary announcement of the first Pan-American Medical Congress, to be held at Washington, D. C., September 5, 6, 7 and 8, 1893.

It is a most interesting pamphlet, neatly gotten up, and shows a world of pains taking labor in its preparation, reflecting much credit on its author, Dr. Chas. A. L. Read. It shows him to be a man possessed of extraordinary ability to organize and skill to execute. It was he who conceived the idea of unifying all American Medicine, and this is the plan by which the object is hoped to be accomplished.

Upon a casual examination the organization appears to be fairly representative, i. e., there seems to be no discrimination, or, rather less discrimination in favor of particular sections than was shown in the preliminary organization of the Ninth International Medical Congress, which, it will be remembered, gave rise to such prolonged and bitter controversy in the medical press at the time. It is sincerely hoped that no one and no State will feel slighted, and that there will be no "kick."

The election of Dr. Wm. W. Pepper, of Philadelphia (by the bye a doctor and not a surgeon), will, we believe, give general satisfaction. He is a strong man and his works have made him popular in the South as well as in the North and East.

We note with satisfaction the names of several Texas physicians in prominent places.

While on this subject we might as well say, this Congress is going to cost something; and the expenses of organization must be paid out of the membership fees, and it is expected that a great deal of this will be paid in advance. Physicians who feel interested in the object and expect to attend, or if unable to be present, would like to have a copy of the proceedings, are requested to register in advance. This can be done by sending the name and address, together with the entrance or membership fee, $10, to Dr. A. M. Owen, the Treasurer, at Evansville, Indiana. Dr. Owen has given a heavy bond (that might seem unnecessary but it is business), and is prepared to register all who are eligible. It would be well to do this, as time and trouble and inconvenience will thereby be saved; it will simply be impossible to register all after the meeting begins.

Dr. Reed will send a copy of this handsome sixty page pamphlet to all who register in advance.

